# A Brief Review on the Role of Vesicular Monoamine Transporter_2_ Inhibitors in Hyperkinetic Movement Disorders

**DOI:** 10.22037/ijcn.v15i3.33144

**Published:** 2021

**Authors:** Ali NIKKHAH

**Affiliations:** 1 Department of Pediatric Neurology, Research Center, Research Institute for Children’s Health, Shahid Beheshti University of Medical Sciences, Tehran, Iran.; 2 Pediatric Neurology Department, Mofid Children’s Hospital, Faculty of Medicine, Shahid Beheshti University of Medical Sciences, Tehran, Iran.

**Keywords:** Vesicular monoamine transporter_2 _(VMAT_2_) inhibitors, Hyperkinetic movements, Dyskinesia, Tetrabenazine, Deutetrabenazine, Valbenazine, Children.

## Abstract

Hyperkinetic movement disorders are a common group of movement abnormalities in children, characterized with repetitive unintended involuntary movements. Major hyperkinetic movements include tremor, tic, dystonia, myoclonus, and chorea. Although a number of drugs have been proven to be beneficial for these abnormalities, some patients may become resistant to conventional treatments. Vesicular monoamine transporter_2 _(VMAT_2_) inhibitors (Tetrabenazine, Deutetrabenazine, and Valbenazine) are new agents introduced in the last decade for treating some of movement disorders, in particular tardive dyskinesia, Huntington chorea, and Tourette syndrome. In this brief review, we discussed the role of these drugs in managing hyperkinetic movement disorders.

## Introduction

Hyperkinetic movement disorders, which are characterized with repetitive unintended involuntary movements, encompass a variety of abnormal movements including tremor, tic, dystonia, myoclonus, and chorea in children (1). Symptomatic children are often treated with conventional drugs. For example, levodopa is an effective agent for some types of dystonia such as dopa responsive dystonia (DRD) (1, 2). However, some patients may fail to appropriately respond to traditional therapeutics. In addition to primary abnormal movements, some children may develop secondary movement disorders due to brain injury, encephalitis, and drugs/toxins. Considering that many of hyperkinetic movement disorders are secondary to a hyper-dopaminergic state in basal ganglia, blocking dopamine receptors by neuroleptics is the most effective and first-line therapy in these conditions. However, these drugs have potentially serious side effects such as Tardive dyskinesia (TD) (3) which is a rare permanent movement disorder due to the prolonged use of dopamine receptor blockers, especially neuroleptics (4). This disorder presents with involuntary movements of the face and tongue, such as continues chewing and tongue protrusion or thrust, along with choreiform movements of extremities (5). 

**Figure 1:**
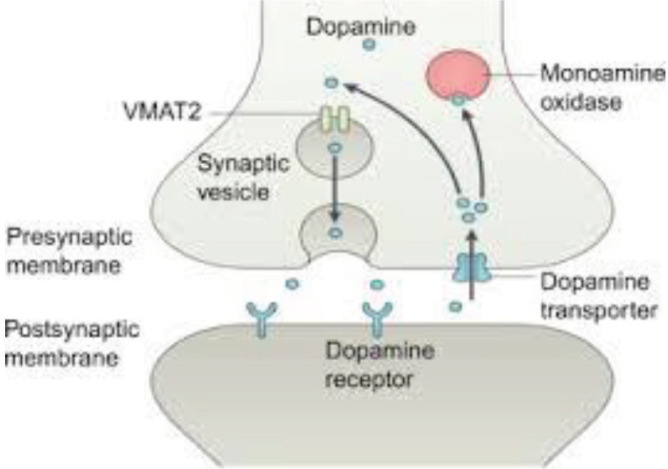
VMAT_2_ (from sciencedirect.com by permission)

Vesicular monoamine transporter 2 (VMAT_2_) is a protein that transports monoamines (especially dopamine) into vesicles in presynaptic neurons (Fig 1).

In the last decade, VMAT_2 _inhibitors (tetrabenazine, Deutetrabenazine, and valbenazine) were presented as a new class of drugs with beneficial effects in the treatment of movement disorders. In 2008, tetrabenazine was approved by the FDA for managing chorea in patients with Huntington disease (HD) (6, 7). There are two types of these transporters: VMAT1, which is present in both the peripheral and central nervous systems, and VMAT2, which has been localized only in presynaptic neurons (8) Also, tetrabenazine has been used as an off-label medication for treating Tourette’s disorder and TD (4). There are a few studies on the application of VMAT_2 _inhibitors in children with hyperkinetic movement disorders. In this brief review, we intend to describe these new drugs and discuss their roles in treating hyperkinetic movement disorders in pediatrics. 

**Drug Description**

**Tetrabenazine (TBZ)** was initially introduced in the 1950s as an antipsychotic agent (6). This drug was the first VMAT_2 _inhibitor approved by the FDA in 2008 to treat HD-induced chorea (7, 8). Tetrabenazine binds to type 2 vesicular monoamine transporters (VMAT_2_) and inhibits the entry of dopamine molecules into these vesicles, depleting their dopamine content. So, the drug is also known as a dopamine depletion agent. The VMAT-2 is found mainly in the central nervous system and transports serotonin, dopamine, norepinephrine, and histamine into vesicles for storage. Tetrabenazine depletes dopamine more selectively compared to other monoamines (9, 10). In the liver, the drug is extensively metabolized to its primary active metabolite (alpha-dihydrotetrabenazine) by the CYP2D6 enzyme. The half-life of alpha-dihydrotetrabenazine is three to eight hours (11, 12). At high doses, TBZ also blocks postsynaptic dopamine receptors (i.e., a dopamine receptor blocker (DRB)) (8). This drug is an oral benzoquinoline derivative and is generally considered to be well-tolerated (13). In different studies, TBZ has been noted to be highly effective for treating HD-associated chorea (13-15). Also, this drug has been effective in the treatment of some other hyperkinetic movement disorders such as tics and dystonia. Tetrabenazine is also used to control motor and phonic tics in children and adolescents with Tourette syndrome (TS). In a relatively old study on ten cases with TS, four patients receiving TBZ showed marked improvement in their tics, and the most common side effect was drowsiness (16). Unfortunately, to date, there is no randomized clinical trial on the efficacy and safety of TBZ in children. The initial dose is 12.5 mg/d which can be then adjusted to 12.5 mg/d every week at the maximum dose of 100 mg/d. Because of its short half-life, TBZ should be given two to three times a day after increasing the dose (11, 12, 14, 15). The most common adverse effects of TBZ in adults are somnolence, mood disorders, and Parkinsonism (14, 17). Drug-induced Parkinson disease is an important side effect limiting the use of TBZ and other similar drugs. Some rare adverse effects of TBZ include transient increased liver enzymes, insomnia, nausea, vomiting, tremor, memory problems, confusion, orthostatic hypotension, dizziness, diarrhea, headaches, hallucinations, paresthesia, pharyngeal spasm and pain, blurred vision, paranoia, and suicide (5, 8, 18). Since 2017, two newer VMAT_2_ inhibitors have been approved by the FDA for managing some hyperkinetic movement disorders such as chorea (especially in patients with HD) and tics (especially in those suffering from TS) (8, 19, 20). 

**Deutetrabenazine (DBZ)** was the first novel medication categorized under VMAT_2 _inhibitors. A chemical substance in DBZ, named deuterium, reduces the drug’s metabolic rate and thus increases its half-life. Deutetrabenazine is an isotopic isomer of TBZ, resulting from the replacement of two methoxy groups (–OCH3) with two trideuteromethoxy groups (–OCD3) at the 9 and 10 positions, which reduces the side effects of DBZ (21). On the other hand, lower serum level fluctuations of this drug reduce its neuropsychiatric side effects such as depression and akathisia (19, 21, 22). Deutetrabenazine is an effective and safe VMAT_2_ inhibitor for treating tardive dyskinesia and HD in adults and adolescents (23). In one open-label trial, DBZ at the doses of 18 to 36 mg/day decreased the severity of tics in adolescents with TS. Irritability, fatigue, and headache were reported as the side effects of DBZ in this study (24). 

**Valbenazine (VBZ)** is the second (after DBZ) new VMAT_2_ inhibitor approved by the FDA since 2017. This drug is an Alpha isomer of TBZ with an adequately long half-life (15-22 hr.) allowing it to be used once daily. The drug is activated by hydrolysis while its deactivation is catalyzed by CYP3A and CYP2D6 in the liver (23). The most common adverse effects of VBZ are sedation, anticholinergic effects, balance disorder, and headache (25). In contrast to TBZ and even DBZ, VBZ has not been associated with increased risk of suicidal thinking and behaviors in adults (27). However, VBZ may increase the QT interval, so it is not recommended to be used by patients with prolonged QT intervals (28). Some randomized clinical trials were conducted on the efficacy and safety of VBZ in adult patients with tardive dyskinesia; almost all of them reported improvements in dyskinetic movements (20). Recently, these drugs, as a second-line option after clonidine, have been used for managing TS. Today, VMAT2 inhibitors are superior to neuroleptics for controlling chronic and severe tics (29, 30). 

## In Conclusion

As mentioned above, VMAT_2_ inhibitors are promising medications for some hyperkinetic movement disorders such as chorea (especially in HD), tics (in TS), and tardive dyskinesia (TD). These drugs are relatively safe and appropriate for long-term usage in patients with TD. There are not enough evidence-based randomized clinical trials on the applicability of these drugs in children and adolescents; however, some experts prescribe TBZ for children with chorea and chronic tics and dyskinesia, who are refractory to conventional therapies. 
